# Vaping discussion in the COVID-19 pandemic: An observational study using Twitter data

**DOI:** 10.1371/journal.pone.0260290

**Published:** 2021-12-08

**Authors:** Joanne Chen Lyu, Garving K. Luli, Pamela M. Ling

**Affiliations:** 1 Center for Tobacco Control Research and Education, University of California, San Francisco, California, United States of America; 2 Department of Mathematics, University of California, Davis, California, United States of America; University of California San Diego School of Medicine, UNITED STATES

## Abstract

**Background:**

With the spread of COVID-19, significant concerns have been raised about the potential increased risk for electronic cigarette (e-cigarette) users for COVID-19 infection and related syndromes. Social media is an increasingly popular source for health information dissemination and discussion, and can affect health outcomes.

**Objective:**

This study aims to identify the topics in the public vaping discussion in COVID-19–related Twitter posts in order to get insight into public vaping-related perceptions, attitudes and concerns, and to discern possible misinformation and misconceptions around vaping in the COVID-19 pandemic.

**Methods:**

Using the tweets ID database maintained by Georgia State University’s Panacea Lab, we downloaded the tweets related to COVID-19 from March 11, 2020, when the World Health Organization declared COVID-19 a pandemic, to February 12, 2021. We used R to analyze the tweets that contained a list of 79 keywords related to vaping. After removing duplicates and tweets created by faked accounts or bots, the final data set consisted of 11,337 unique tweets from 7,710 different users. We performed the latent Dirichlet allocation (LDA) algorithm for topic modeling and carried out a sentiment analysis.

**Results:**

Despite fluctuations, the number of daily tweets was relatively stable (average number of daily tweets = 33.4) with a sole conspicuous spike happening on a few days after August 11, 2020 when a research team published findings that teenagers and young adults who vape face a much higher risk of COVID-19 infection than their peers who do not vape. Topic modeling generated 8 topics: linkage between vaping and risk of COVID-19 infection, vaping pneumonia and the origin of COVID-19, vaping and spread of COVID-19, vaping regulation, calling for quitting vaping, protecting youth, similarity between e-cigarette or vaping-associated lung injury (EVALI) and COVID-19, and sales information. Daily sentiment scores showed that the public sentiment was predominantly negative, but became slightly more positive over the course of the study time period.

**Conclusions:**

While some content in the public discourse on vaping before the COVID-19 pandemic continued in Twitter posts during the COVID-19 time period, new topics emerged. We found a substantial amount of anti-vaping discussion and dominantly negative sentiment around vaping during COVID-19, a sharp contrast to the predominantly pro-vaping voice on social media in the pre-COVID-19 period. Continued monitoring of social media conversations around vaping is needed, and the public health community may consider using social media platforms to actively convey scientific information around vaping and vaping cessation.

## Introduction

Coronavirus disease 2019 (COVID-19) is a contagious disease caused by severe acute respiratory syndrome coronavirus 2 (SARS-CoV-2) [1]. Since COVID-19 was declared a pandemic by the World Health Organization (WHO) on 11 March 2020, there have been 108,733,129 confirmed cases globally as of February 12, 2021, and the United States has reported the highest number of confirmed COVID-19 cases [2]. Smoking cigarettes has been associated with increased severity of COVID-19 disease [3]. In addition, significant theoretical concerns have been raised about the potential increased risk among electronic cigarette (e-cigarette) users for COVID-19 infection and syndromes [4,5], although few studies specifically address e-cigarette use and COVID-19 outcomes.

Coronaviruses are respiratory infections [6], and use of e-cigarettes (colloquially known as "vaping") has been associated with pulmonary inflammation in response to infection [7,8]; therefore, e-cigarette users may be at increased risk of contracting COVID-19 [8,9]. In a 2020 cross-sectional study among adolescents and young adults, COVID-19 diagnosis was 5 times more likely among ever-users of e-cigarettes compared with non-users [10]. In addition, vaping may impair immune response to viral infection, which may increase risk for many severe COVID-19 disease [4,11]. Therefore, these mechanisms suggest that those who vape may be more susceptible to pulmonary complications following a COVID-19 infection [6]. However, researchers have also hypothesized that cannabidiol (CBD), which can be administered via vaping, has the potential to limit the severity and progression of COVID-19 symptoms [12]. Meanwhile, unsubstantiated health claims about vaping circulated online in the early period of the pandemic. For example, one study described comments that vape devices would increase humidity in the lungs and thereby prevent COVID-19 infections; and that propylene glycol (PG), a common ingredient in e-cigarettes, is able to destroy harmful COVID-19 airborne contagions [13].

Social media are increasingly preferred sources for health information dissemination and discussion [14] and can affect health outcomes [15,16]. However, social media-based research studying the public discourse on vaping in the COVID-19 pandemic has been scant. Two studies focusing on both vaping and COVID-19 analyzed social media data before the end of April, 2020 [17,18]. WHO declared the COVID-19 outbreak a global pandemic on March 11, 2020. This declaration was found to have a significant impact on public awareness and behavior [19]. However, there has been lack of understanding of the public discourse at the intersection of COVID-19 and vaping when the COVID-19 pandemic unfolded in full swing. Continued surveillance of social media data and monitoring of public discourse in this line are needed as the pandemic progresses [13]. To fill the gap, this study analyzed the public vaping discussion in COVID-19–related Twitter posts from March 11, 2020 through February 12, 2021 to identify the topics and sentiments from tweets around e-cigarettes and vaping. Understanding the public discourse on e-cigarettes and vaping and COVID-19 will not only shed light on public vaping-related perceptions, attitudes and concerns during a large-scale public health crisis, but also enable identification of possible misinformation and misconception around vaping in the COVID-19 pandemic. Therefore, this study will offer insights for vaping-related scientific knowledge promotion and interventions for curbing the spread of inaccurate information, which is especially crucial during the COVID-19 pandemic, an unprecedented respiratory disease crisis.

## Method

### Data extraction and preprocessing

We downloaded the IDs from the website maintained by Georgia State University’s Panacea Lab [20], obtaining a total of 3,414,483 tweets, without retweets, from March 11, 2020 through February 12, 2021. These tweets were collected daily by the Panacea Lab using the following 13 keywords related to Covid-19: COVD19, CoronavirusPandemic, COVID-19, 2019nCoV, CoronaOutbreak, coronavirus, WuhanVirus, covid19, coronaviruspandemic, covid-19, 2019ncov, coronaoutbreak, and wuhanvirus. Since the Panacea Lab can provide only the IDs for the tweets, we need to convert the IDs back to the original tweets using the *Hydrator* software [21]. These data were collected using publicly available resources only and were accessed in compliance with Twitter terms of use.

During the data preprocessing stage, we used the *gsub* function in R (The R Foundation) to keep tweets whose language field was specified as English. We performed all text mining using RStudio Version 1.4.1103. Next, all the tweets were converted to lowercase. Referring to previous studies examining Twitter posts about e-cigarettes [22,23], we used a list of 79 search keywords to extract vaping-related tweets, including general e-cigarette terms and their variants (e.g., e-cigarette, e-cig, e-juice, e-liquid, vaper, electronic cigarette, e-vaporizer, e-hookahs), e-cigarette brand names (eg, blu, JUUL, NJoy, green smoke, Krave), and e-cigarette use (eg, vaping, e-smoke, vapenation). After obtaining the relevant tweets for our analysis, we prepared two batches of tweets, one for text mining and the other for sentiment analysis. For text mining and sentiment analysis, the data has to be processed differently. Specifically, for sentiment analysis, we converted all the emojis to words, whereas for text mining we removed all the emojis since they do not provide essential information. Next, we created a script to remove the URLs, mentioned names, non-ASCII (American Standard Code for Information Interchange) characters, and all characters other than English letters or spaces (eg, “1,” “?,” etc). Duplicated tweets were removed using the R package *dplyr*, version 1.0.2. In order to filter tweets created by faked or bots accounts, we removed tweets that were almost identical. This is done using the document-term matrix (DTM) consisting of rows that correspond to the tweets and columns that correspond to the terms to identify similar terms in each tweet (see S1 Appendix for details). For tweets that were 80% similar, we retained the most representative one (i.e., the longest tweets in terms of the number of words). Furthermore, we used the *tweetornot* Version 0.1.0 package to remove users identified as bots with a 95% probability or higher.

**Fig 1:**
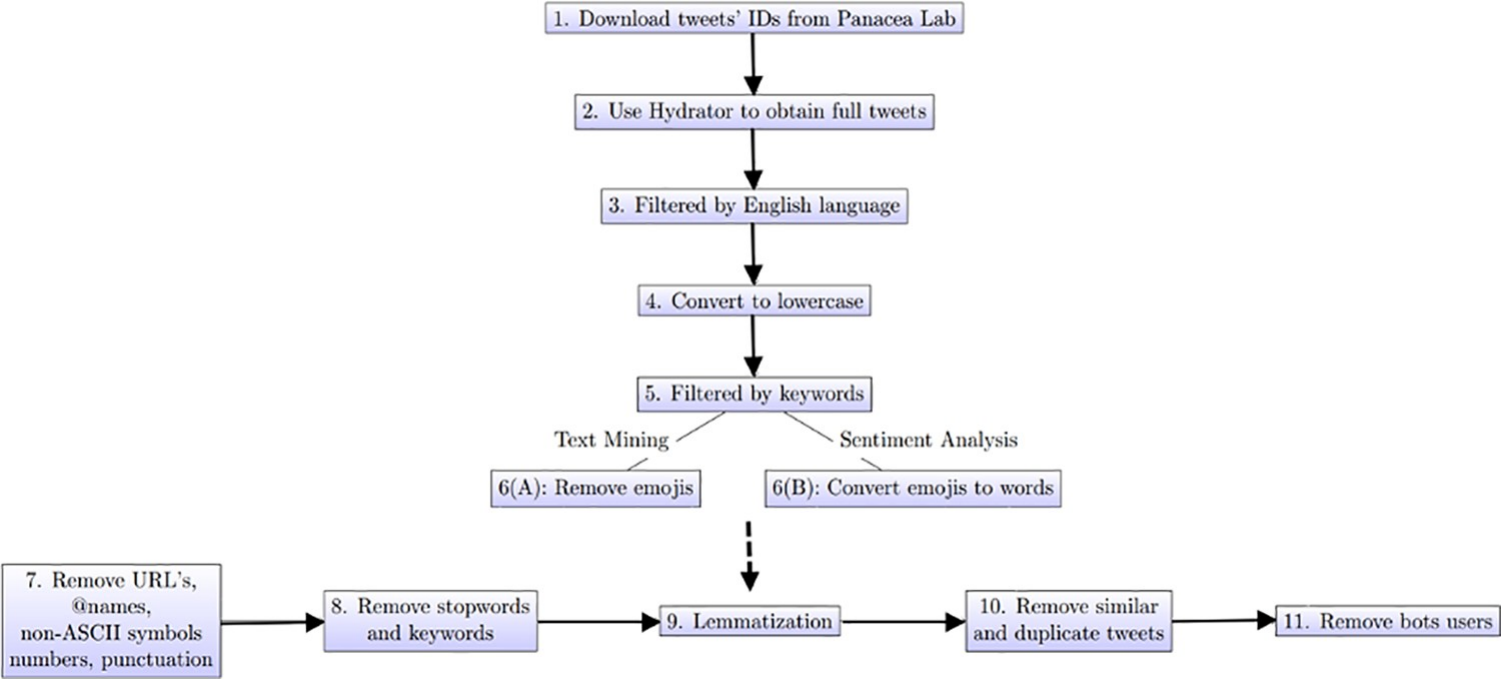
Data extraction and preprocessing procedure.

The final cleaned data set consisted of 11,337 unique tweets from 7,710 different users. We further cleaned the tweets by removing the stopwords, i.e., words and characters that were of little or no analytical value (e.g, “a,” “and,”,”are”, “&,” etc). We performed this task by creating our own list of stop words by appending the 13 keywords related to “COVID19” and the 79 keywords related to “vaping” to the standard English stop words list from the R package *tidytext*, version 0.2.6. The reason to add the keywords to the stopwords is that since every tweet would contain one or more of those keywords and having them in the tweets does not contribute to further our understanding of the tweets. Lastly, we stemmed and lemmatized the words to their root forms using the R package *textstem*, version 0.1.4 (e.g., *smoking*, *smokes*, and *smoked* were converted to *smoke*). Fig 1 summarizes our data extraction and preprocessing procedure.

### Topic modeling and sentiment analysis

Topic modeling is an automatic way of summarizing a large collection of documents. We can use it to discover hidden themes, group documents into the discovered themes, and summarize the documents by topic. Topic modeling is often referred to as *soft* clustering, but it is more robust and provides better and more realistic results than hard clustering methods (such as k-means clustering) [24]. A hard clustering algorithm assumes a distance measure between topics and assigns one topic to each document, whereas topic modeling assigns a document to a collection of topics with different probabilities without any assumption on the distance measure between topics. There are many topic models available. The most popular one for topic modeling is the Latent Dirichlet Allocation (LDA) model [24], developed by David Blei, Andrew Ng, and Michael I Jordan [25].

**Fig 2:**
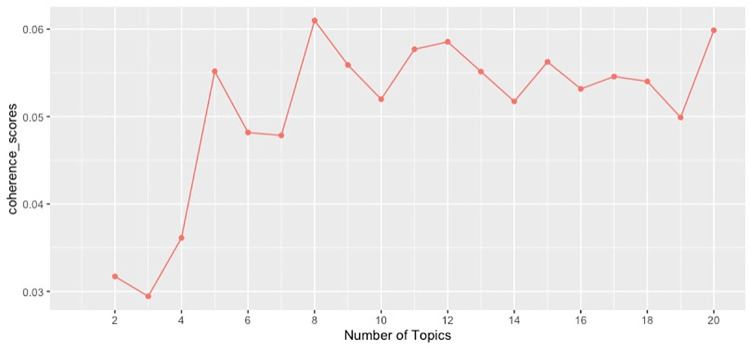
Coherence scores for different numbers of topics.

To extract common topics, we performed the LDA algorithm on the cleaned tweets. This task is done using the R *textmineR* package, version 3.0.4. To run the LDA algorithm one needs to input the number of expected topics. Since the number of topics is unknown to begin with, we need to run the LDA algorithm for a range of different topic numbers and choose one that maximize the coherence score. The LDA algorithm was run on the data for topic numbers from 2 through 20. For each topic number, we calculated the coherence score using the *textmineR* package. Based on the coherence score plot, we chose 8 topics for the final model, since the topic number = 8 yielded the highest coherence score (Fig 2). We also implemented the interpolation algorithm to plot the distribution of the quantity of daily tweets about each topic [26].

We extracted the top eight terms from each of the 8 topics. For each tweet, the LDA algorithm assigned a (conditional) probability p(topic *i* | tweet *d*) to each of the 8 topics. The topic with the highest probability is assigned a tweet. Afterward, the tweets are grouped according to the most prevalent topics. Representative tweets for each topic were obtained by randomly sampling 100 tweets from each topic. Two of the authors then independently examined the sampled tweets. Through a group discussion, the authors select the most representative ones. If one of the authors thought that there were no conspicuous topics that emerged from the first 100 sampled tweets, another 100 tweets would be sampled and further reviewed. This process continued until the two authors reached consensus on a clear common topic. To facilitate the topic assignment, we used the *textmineR* package’s topic label function to generate an initial set of topics. After carefully reading through the sampled tweets from each topic, the authors refined the machine-generated labeling to give each topic a concise description. Sentiment analysis assigns a score to a document to indicate whether the expressed opinion is positive, negative, or neutral. The *syuzhet* package is the most popular and efficient R package for sentiment analysis [27]. Since we are interested in the evolution of the sentiments over a period of time, we will choose methodologies that can quantify the sentiments. *Syuzhet* particularly suits our purpose as it assigns continuous values for the sentiments and it allows us to quantitatively compare the texts, whereas other methods such as StandfordNLP and VADER use categorical values (i.e., good, neutral, or negative, etc).

## Results

**Fig 3:**
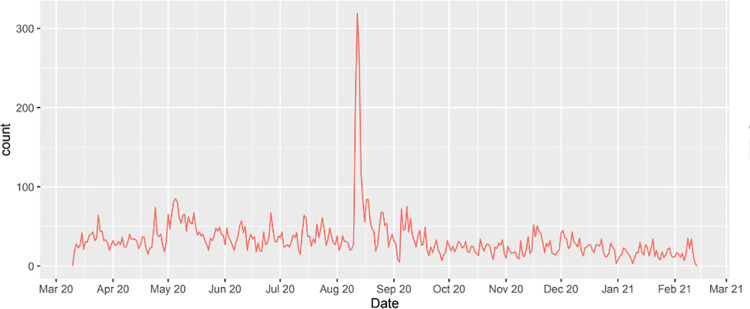
Distribution of the quantity of daily tweets, March 11, 2020—February 12, 2021.

After data extraction and preprocessing as described in the methods section, a total of 11,337 tweets from 7,710 different users were included in the analysis. The time frame of the data was from March 11, 2020 to February 12, 2021. As shown in Fig 3, despite fluctuations, the number of daily tweets was relatively stable (average number of daily tweets = 33.4), with a sole conspicuous spike happening on a few days after August 11, when the first study examining connections between youth vaping and COVID-19 was published online [10]. This study found that teenagers and young adults who vape face a much higher risk of COVID-19 than their peers who do not vape. Discussion on this study immediately became the focus of vaping discussion that day and set the record for the highest number of tweets in a single day on August 12, 2020 (n = 319).

### Topic modeling

**Fig 4:**
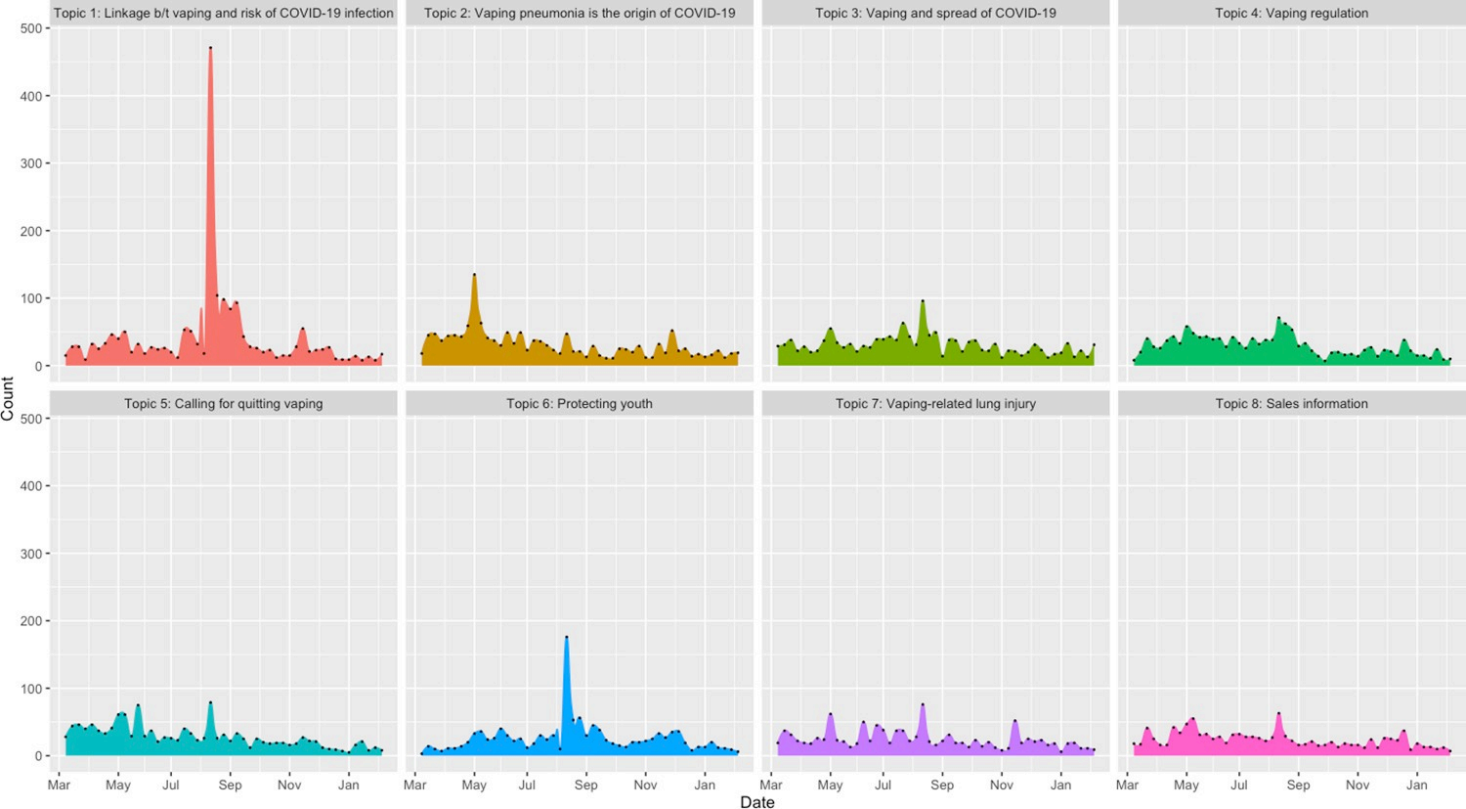
Distribution of the quantity of daily tweets about each topic, March 11, 2020—February 12, 2021.

**Fig 5:**
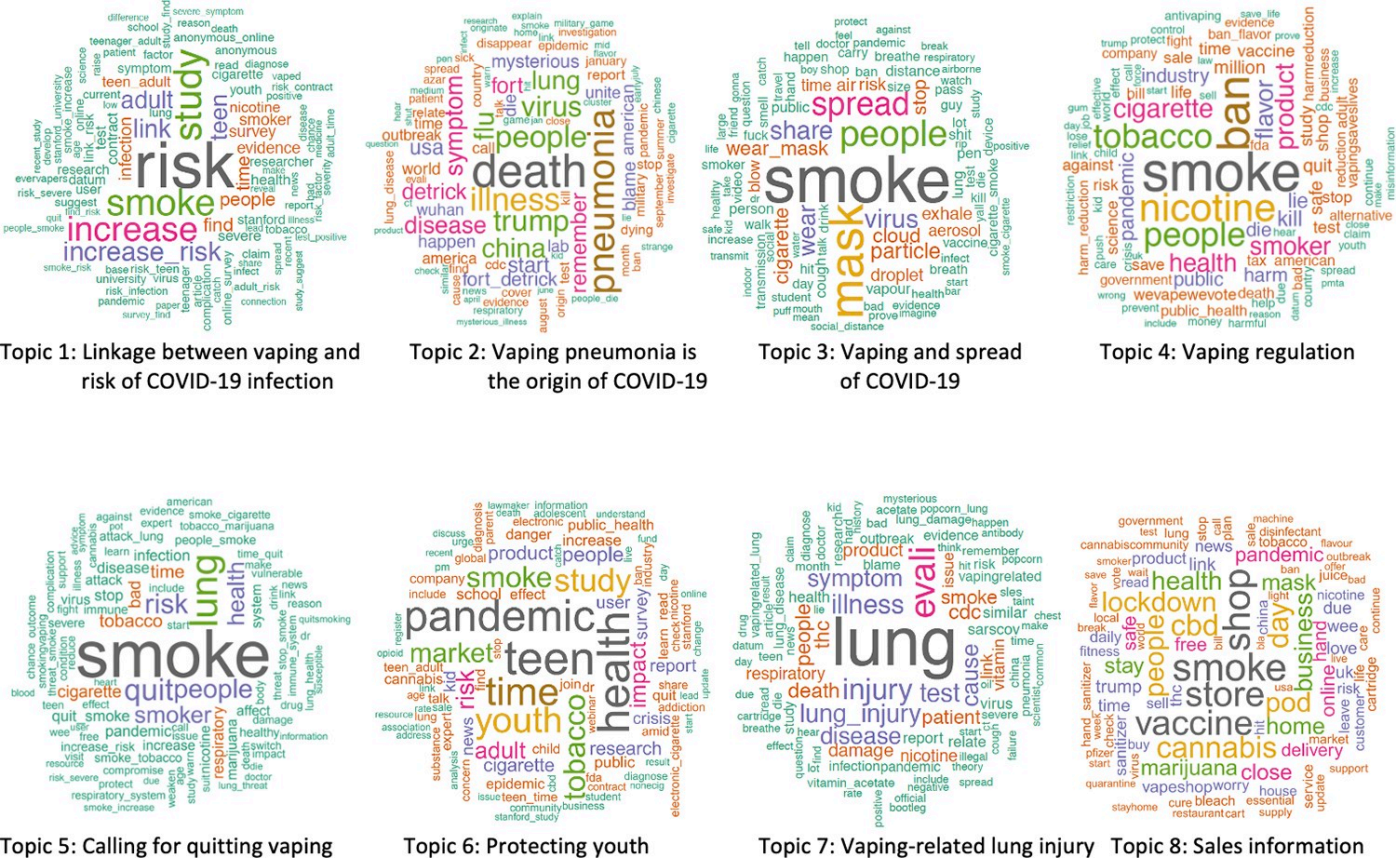
Word clouds showing each topic’s 50 most frequently occurring words contributing to topic model.

Eight topics were generated from the public vaping discussion in COVID-19–related Twitter posts. According to the quantity of tweets in descending order, the eight topics were: Topic 1 linkage between vaping and risk of COVID-19 infection, including 1,937 tweets and accounting for 12.16% of the total tweets (i.e., 11,337) included in the analysis; Topic 2 vaping pneumonia and the origin of COVID-19 (1,513 tweets, 13.35%); Topic 3 vaping and spread of COVID-19 (1,501 tweets, 13.24%); Topic 4 vaping regulation (1,426 tweets, 12.58%); Topic 5 calling for quitting vaping (1,367 tweets, 12.06%); Topic 6 protect youth (1,246 tweets, 10.99%); Topic 7 similarity between EVALI and COVID 19 (1,184 tweets, 10.44%); and Topic 8 sales information (1,163 tweets, 10.26%). Fig 4 shows the temporal pattern in the number of each topic’s daily tweets during the study timeframe. Basically, the number of daily tweets about each topic was stably under 100, and discussion on the Gaiha study [10] that found vaping linked to COVID-19 risk in teens and young adults brought peak daily tweets for most topics around August 11, 2020, when the study was published online. Topic 2 (vaping pneumonia is the origin of COVID-19) was the only exception, with the peak happening around the beginning of May 2020. The word clouds in Fig 5 showed the weights of the top 50 terms for each topic. Within a topic, terms with larger weights display in larger font sizes. Terms with approximately the same weight display in the same color. Description of the eight topics, together with two representative tweets for each topic (selected as described in methods), are elaborated below.

#### Topic 1: Linkage between vaping and risk of COVID-19 infection

This topic focused on the linkage between vaping and risk of contracting COVID-19. Though there had been discussion about whether vaping raises chances of coronavirus infection and death, the study titled *Association Between Youth Smoking*, *Electronic Cigarette Use*, *and COVID-19* published on August 11 pushed this topic to the peak (Fig 4). While most tweets forwarded the research findings and emphasized the increased risk of getting infection with COVID-19 among young people found by the Gaiha study [10], there were some tweets questioning the rigor of the study, such as "anonymous online survey" and "weak evidence". Some representative tweets were:

*“#vaping #coronavirus vaping linked to COVID-19 risk in teens and young adults. Teenagers and young adults who vape face a much higher risk of #COVID19 than their peers who do not vape. [link]” (8/11/2020)*

*“@childrensmn you are spreading #COVID19 misinformation. The @stanfordtpt study that claimed this ’risk,’ in a press release, was an anonymous online survey. Read it. They actually found no difference in infection risk between vapers and never-vapers among those tested. #junkscience” (12/9/2020)*

#### Topic 2: Vaping pneumonia is the origin of COVID-19

This topic centered around the conspiracy theory around vaping and COVID-19 origin. Tweets mentioned the similar symptoms and CT images of COVID-19 and the “vaping pneumonia”, and discussed that US was the actual origin of COVID-19 but blamed China, and suggested that “vaping pneumonia” cases actually included COVID-19 cases. Tweeters also questioned the "coincidence" that U.S. Army Medical Research Institute of Infectious **Diseases** labs at Fort Detrick was shut down by CDC and the outbreak of vaping pneumonia followed closely.

*“@[tag] @washingtonpost then Fort Detrick was shut down by CDC in August. Later in September the so-called vaping pneumonia cases came out. Those cases have much similar symptoms like COVID19. So all these things are just coincidence? [link]” (5/7/2020)*

Generally speaking, tweets under this topic featured a doubtful tone and were posed as questions, such as “how do you justify the e-cigarette disease or influenza in America last year are not coronavirus?” (4/18/2020), and “why these pneumonia cases only happened in US and why only started from 2019 September?” (5/6/2020). One of the representative tweets was:

*“@Reuters apparently so-called mainstream media is lying to westerners! Don’t be fooled! Origin was from US, google electronic vaping lung. Vaping is so popular all around the world, but only the US has so much case of vaping lung which are same as the COVID-19. Too much hidden by us!” (1/19/2021)*

#### Topic 3: Vaping and spread of COVID-19

This topic was around the spread of COVID 19. Tweets under this topic discussed the effectiveness of wearing mask to prevent the spread of COVID-19 usually by comparing the diameters of COVID particles and vape particles, whether vaping aerosol transmits coronavirus, and if sharing vape devices will lead to spread of covid-19, and therefore should be avoided. Representative tweets were:

*“Do masks work? Vape smoke is 2.5 microns, and #covid19 is between 0.15–0.25 microns. You decide for yourself if masks work to stop inhaling or exhaling #coronavirus droplets. [link]” (7/26/2020)*

*“Potentially infected respiratory droplets are not only expelled with each cough, but also carried in each exhale of cigarette or vape smoke. This means that if you can smell the smoke, you might be in a coronavirus danger zone, doctors say. [link]” (11/11/2020)*

#### Topic 4: Vaping regulation

The vaping regulation topic mainly opposed regulation of the e-cigarette industry. Besides the opinion expressed—that COVID-19 was exploited to ban vaping—two points were frequently mentioned to oppose e-cigarette regulation: one emphasized the harm reduction effect of e-cigarettes. "Safe alternatives" was a frequently mentioned term in tweets of this kind; and hashtags such as #harmreduction and #vapingsaveslives were frequently used. The second point emphasized e-cigarette industry and vape stores’ donations and provision of supplies to support fight with COVID-19, and further pointed out that the regulation would harm the vape industry. Representative tweets were:

*“Stop banning vaping products!!!*

*Why policymakers are wrong to use the coronavirus crisis to ban vaping | the national interest [link]” (5/18/2020)*

*“Buried in the new #COVID19 bill on page 5,136 is a provision unjustly targeting the #vaping community. Banning the USPS from shipping #vape products will make it harder for smokers to quit and harm small businesses[link]” (12/22/2020)*

#### Topic 5: Calling for quitting vaping

The focus of this topic was calling for vapers to quit. Tweets under this topic urged vapers to quit by emphasizing that vaping compromises the respiratory system, makes lungs more vulnerable, and therefore increases the risk of COVID-19 infection, or advocated to reduce risk of serious lung disease by the virus by quitting smoking and vaping. A certain number of tweets included the Quitline information, such as the phone number and websites. Representative tweets were:

*“Because it attacks the lungs, the #coronavirus could be a serious threat to those who vape or smoke tobacco or marijuana.*

*Call the Massachusetts smokers’ helpline at 1-800-quit now (1-800-784-8669) for free coaching and support 24/7. Learn more at [link][link]” (4/13/2020)*

*“There has never been a better time to quit. using tobacco (vaping/smoking) weakens your immune system and can make lung illnesses, like #COVID19 worse. Get help today! Call 1-800-quit-now (1-800-784-8669) or visit [link]. #greatamericansmokeout #quitsmokingtoday [link]” (11/19/2020)*

#### Topic 6: Protecting youth

A portion of tweets under the topic of protecting youth mentioned the COVID impact on e-cigarette and vape market, and that the e-cigarette industry exploited the COVID-19 to market products targeting youth, such as using pandemic-themed ads to boost sales. Tweets also highlighted that it was time to take action to protect young people. Information about prevention and intervention resources was provided in some tweets, including resources for parents. Representative tweets were:

*“Congressional lawmakers say puff bar exploited the coronavirus pandemic to sell its products to schoolchildren. To read the full article, go to [link]. For youth vaping prevention resources, visit our website at [link][link].” (6/8/2020)*

*“More than one in four high school students already vape—and at a time when young people are navigating an extremely difficult back-to-school season amid the covid-19 pandemic, it’s more important than ever for parents to talk to their kids about vaping. [link]” (10/2/2020)*

#### Topic 7: Vaping-related lung injury

Tweets under this topic discussed the similar symptoms between e-cigarette or vaping-associated lung injury (known as EVALI) and COVID-19. Though while tweets like "I cannot stop thinking about how the symptoms of covid-19 and vape related lung failure corelate so closely" (3/24/2020) seem similar to Topic 2, the difference is that this topic did not include conspiracy theories such as “cover up” and shifting blame to China, instead, tweeters reminded others to report use of vaping products to doctors when people show COVID-19 symptoms to facilitate accurate diagnosis. Representative tweets were:

*“#COVID19 symptoms can be similar to those of e-cigarette or vaping-associated lung injury, known as #EVALI. Be sure to report use of #vaping products to your doctor during the COVID19 #pandemic. EVALI put 8 California #patients in #hospital in April. [link]” (7/1/2020)*

*“In late 2019, a vaping-related illness named EVALI began to surface. Given that its symptoms mirror those of COVID-19, it can be difficult to tell them apart. #covid19 #vaping [link]” (10/1/2020)*

#### Topic 8: Sales information

This topic was around sales information, introduction of new products, new flavors, and restocking. Under the lockdown, most bricks and mortar shops as non-essential business were shut down. Many tweets provided websites of online stores and emphasized fast delivery. In addition, a small portion of tweets with sales information used hashtags to promote purported benefits of vaping, such as #depress, #support, #peace. Representative tweets were:

*“Get your favourite relx flavors at [link] online store for all your vaping needs. [link]*

*#vape #vapeworld #vapecommunity #covid19 #elqiuids #delivery #notobacco #breakthehabbit [link]” (5/22/2020)*

*“Our CBD JUUL’s pods have zero odor of cannabis and zero leaks! If you haven’t tried watermelon flavor yet, we do recommend it. visit us: [link] #juulspods #cbdcomlaranja #bateriajuuls #covid19 #coronavirus #immuneboosters #flusymptoms [link]” (1/30/2021)*

### Sentiment analysis

**Fig 6:**
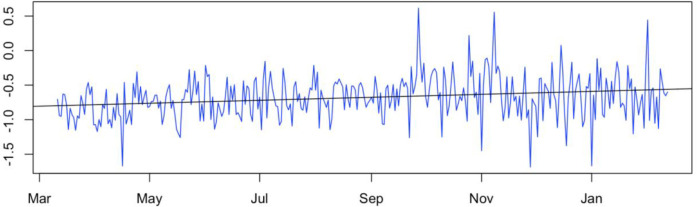
Distribution of daily average sentiment scores, March 11, 2020—February 12, 2021.

Daily sentiment scores showed that the public sentiment in the time of our examination (338 days in total) was predominantly negative toward vaping except three days with the average sentiment being -.68 (SD = .31), ranging from -1.69 to .61. Even though generally negative, the public sentiment shown in vaping discussion in COVID-19–related Twitter posts has been slightly improving from March 11, 2020 to February 12, 2021, with the best linear fit slope = 7.035e-04 (with statistically significant p-value = 4.39e-05) (Fig 6).

## Discussion

### Principal findings

Through analysis of eleven months of Twitter data from March 11, 2020 to February 12, 2021, this study found several topics emerged from the public discussion on vaping in their COVID-19-related posts. Some of the topics we identified were similar to public vaping discussions reported in the literature before the COVID-19 pandemic, such as harms from vaping and vaping regulation [18,23]. However, we found that during the pandemic, these topics were further linked with COVID-19. For example, tweets mentioning harms from vaping stressed that vaping compromises the lung and respiratory system and further increased risk of COVID-19 infection [13]. Past studies of Twitter data prior to the COVID-19 pandemic found negative or mixed reactions to e-cigarette regulation dominated the public discourse [28]. In our study during the pandemic, tweeters opposing vaping regulations claimed that policymakers took advantage of the COVID-19 crisis to ban vaping (Topic 4). Findings of this study are consistent with findings of early analyses of public vaping-related discourse on social media from the beginning of the pandemic till the end of April 2020, where discussion about shutting down vape stores [18] and transmission of coronavirus by sharing vapes was identified [13]. However, the main finding of the Janmohamed study [18], emergence of discourse around vape-administered CBD treatment for COVID-19, was not identified in this study. This may be because that majority of the data analyzed by the Janmohamed study were blogs, and information spread on different media platforms is different. Another possible reason is that as the pandemic progressed, discourse about vape-administered CBD treatment for COVID-19 gradually decreased. Tweets mentioning CBD appeared mainly in the sales information topic in this study. In addition, one thing in common with the pre-COVID-19 twitter analysis is that significant events can greatly stimulate discussion. The most obvious example of event-based discussion was the publication of the Gaiha study [10], which had the biggest numbers of daily tweets during our study.

Another important finding of this study is that the public discourse on vaping in the COVID-19-related tweets was not overwhelmingly pro-vaping. This differs from the prior literature: a 2019 published scoping review of messages presented in e-cigarette discussion on multiple social media platforms including Twitter concluded that the social media had been dominated by pro-vaping messages disseminated by the vaping industry and vaping proponents [29]. Though pro-vaping content, such as advocating harm reduction and opposing vaping regulations existed in public discussion in our Twitter data, anti-vaping voices were conspicuous. A considerable number of tweets discussed the harm from vaping on lungs, and pointed out this would increase the risk of COVID-19 infection and complications. Tweets involving the negative impact of vaping on adolescents and young adults, and protecting the young population during the pandemic and when youth return to school after lockdowns accounted for over 10% of the tweets in our analysis. We also found the topic of calling for quitting vaping emerged in the public vaping discourse, and information of Quitline, educational websites, and webinars were provided in many tweets. In contrast, pre-COVID-19 studies found that Twitter was heavily exploited by the e-cigarette industry, and that tweets related to e-cigarettes were overwhelmingly advertising and commercial [22,30], which lead to findings that vaping discussion was dominated by pro–e-cigarette content [23]. Though this study did not analyze the source of each piece of content, the existence of anti-vaping voices in our data suggests that sources other than the e-cigarette industry have begun to utilize Twitter to express themselves.

Analysis of public discourse on social media provides an opportunity to understand the information that the public is inadvertently exposed to, which may help to explain health-related behavior and offer insight for public health interventions. There have been various conspiracy theories about COVID-19 [31]. When it came to the intersection of COVID-19 and vaping, we found that while the similarity of symptoms between COVID-19 cases and EVALI were mentioned as evidence supporting conspiracy theories that the US was the origin of COVID-19 and covered up early COVID-19 infections by labeling them “vaping pneumonia,” the symptom similarities were also used to remind people to tell doctors about their e-cigarette use in order to assist with diagnostic accuracy. Social media have played a large role in the spread of conspiracies [32]. Therefore, learning how to use social media platforms to quickly identify the conspiracy theories and effectively correct misinformation should be an area that deserves more efforts in the future public health crises. This study also found that the harm reduction discourse around vaping still existed in the pandemic as in the pre-COVID-19 period. To cope with the stress and anxiety in the crisis, many used tobacco and alcohol as coping strategies [33]. Therefore, the harm reduction concept may potentially lead people to vaping to alleviate stressful emotion in the pandemic. In addition, we found Twitter contained information that may significantly influence efforts to combat COVID-19, such as the usefulness of wearing masks to prevent COVID-19 infection. Surveillance of social media discussions would help identify needs for information or recommendations that health authorities should aim to address. Last but not least, the predominantly negative sentiment about vaping in tweets in our study was consistent with survey findings among young vapers that reported 57% worry that their vaping puts them at risk of serious illness from COVID-19 and 62% indicate more interest in quitting vaping now compared to before the pandemic [34]. Taken together, these data suggest that the pandemic is an opportunity to support quitting vaping. The improving sentiment about vaping also suggests it is important to act quickly, seize the opportunity to increase the public awareness of vaping harm, and urge more people to quit.

### Limitations

This paper studied Twitter data to understand of the public vaping and COVID-19 discussion on social media. Though Twitter is one of the most popular social media platforms and widely used as the social media data source in academic work [17,18,23,28,35], it is not the only one. Therefore, to fully understand the public discussion about vaping, social media data from more sources should be included. Second, this study focused on the content of Twitter posts and did not examine the characteristics of the Twitter users who were the producers and posters of the content. Knowing the characteristics of posters of certain topics may illuminate some of the dissenting voices we found, to not only deepen our understanding of the dynamics and motivation behind the topics, but also provide more insight into future monitoring and regulation.

### Conclusions

Analysis of vaping discussion in COVID-19-related Twitter posts provided a unique opportunity to understand public opinion around vaping in the pandemic. While the public discourse on vaping in the COVID-19 Twitter posts shared common content with that before the COVID-19 pandemic, distinction between the two periods was obvious and more COVID-19 related topics emerged. In contrast to prior studies that found a predominantly pro-vaping voice in the pre-COVID-19 period, we found a noticeable anti-vaping discourse and the public sentiment around vaping was predominantly negative in the tweets during the time period of our study. This study highlights the need to continue monitoring social media conversations as they evolve, in order to provide timely interventions that may influence health outcomes and the success of addressing the COVID-19 pandemic. The public health community may need to consider using more social media platforms to correct misinformation and actively convey scientific information to around vaping and vaping cessation.

## Supporting information

S1 Appendix (DOCX)
