# Supplementary material for: Vaping discussion in the COVID-19 pandemic: An observational study using Twitter data
Source: PLoS One. 2021 Dec 8;16(12):e0260290. doi: 10.1371/journal.pone.0260290 (PMC8654216; doi:10.1371/journal.pone.0260290)
Supplement: S1 Appendix — (DOCX) [file pone.0260290.s001.docx]

Each entry in the DTM denotes the number of times a term appears in a tweet. The similarity matrix $S=[S_{ij}]$, which measures how similar the *i*-th tweet is to the *j*-th tweet, is obtained by computing the dot product $S_{ij}=\frac{{|R}_{i}\cdot R_{j}|}{\|R_{i}\|\|R_{j}\|}$ between the *i*-th row vector $R_{i}$ and the *j*-th row vector $R_{j}$in the DTM, which represents the cosine of the angle between the row vectors $R_{i},R_{j}$. The number $S_{ij}$ roughly corresponds to the percentage of terms common to the *i*-th and *j*-th tweets. Therefore, if the *i*-th and the *j*-th tweets are identical, then $S_{ij}=1$; if they are completely different, then $S_{ij}=0$.
